# Vasoactive intestinal peptide blockade suppresses tumor growth by regulating macrophage polarization and function in CT26 tumor-bearing mice

**DOI:** 10.1038/s41598-023-28073-6

**Published:** 2023-01-17

**Authors:** Wararat Kittikulsuth, Daisuke Nakano, Kento Kitada, Toru Uyama, Natsuo Ueda, Eisuke Asano, Keiichi Okano, Yoko Matsuda, Akira Nishiyama

**Affiliations:** 1 Department of Pharmacology, Faculty of Medicine, Kagawa University, 1750-1 Ikenobe, Miki, Kita, Kagawa 761-0793 Japan; 2 Department of Biochemistry, Faculty of Medicine, Kagawa University, Kagawa, Japan; 3 Department of Gastroenterological Surgery, Faculty of Medicine, Kagawa University, Kagawa, Japan; 4 Oncology Pathology, Department of Pathology and Host-Defense, Faculty of Medicine, Kagawa University, Kagawa, Japan

**Keywords:** Colorectal cancer, Cancer immunotherapy

## Abstract

Macrophages are a major population of immune cells in solid cancers, especially colorectal cancers. Tumor-associated macrophages (TAMs) are commonly divided into M1-like (tumor suppression) and M2-like (tumor promotion) phenotypes. Vasoactive intestinal peptide (VIP) is an immunoregulatory neuropeptide with a potent anti-inflammatory function. Inhibition of VIP signaling has been shown to increase CD8^+^ T cell proliferation and function in viral infection and lymphoma. However, the role of VIP in macrophage polarization and function in solid tumors remains unknown. Here, we demonstrated that conditioned medium from CT26 (CT26-CM) cells enhanced M2-related marker and VIP receptor (VPAC) gene expression in RAW264.7 macrophages. VIP hybrid, a VIP antagonist, enhanced M1-related genes but reduced *Mrc1* gene expression and increased phagocytic ability in CT26-CM-treated RAW264.7 cells. In immunodeficient SCID mice, VIP antagonist alone or in combination with anti-PD-1 antibody attenuated CT26 tumor growth compared with the control. Analysis of tumor-infiltrating leukocytes found that VIP antagonist increased M1/M2 ratios and macrophage phagocytosis of CT26-GFP cells. Furthermore, *Vipr2* gene silencing or VPAC2 activation affected the polarization of CT26-CM-treated RAW264.7 cells. In conclusion, the inhibition of VIP signaling enhanced M1 macrophage polarization and macrophage phagocytic function, resulting in tumor regression in a CT26 colon cancer model.

## Introduction

Colorectal cancer is the third highest ranking cancer type in terms of incidence and the second leading cause of death from cancer worldwide. According to new estimated cases in 2020, there are approximately 940,000 colorectal cancer-related deaths annually^1^. Studies have suggested that immune cells in the tumor microenvironment (TME) may predict patient survival because they play a critical role in controlling tumor progression^2,3^. Macrophages are the major type of tumor-infiltrating leukocytes in solid tumors, including colorectal and lung cancer^4^. Tumor-associated macrophages (TAMs) can be divided into M1-like and M2-like phenotypes depending on their ability to secrete cytokines and chemokines. Moreover, M1-like TAMs contain high class II major histocompatibility complex (MHCII) and act as antigen-presenting cells to activate T cell function against cancers. However, as tumors progress, tumor cells release numerous cytokines to promote M2-like TAM polarization. In turn, M2-like TAMs release anti-inflammatory cytokines and growth factors to assist tumor growth^5,6^. In clinical settings, an increase in M2-like TAMs is associated with a reduction in patient survival^3,5,7^. Furthermore, a high ratio of M1/M2 macrophages in the TME is correlated with a better prognosis in colon cancer patients^8,9^. Thereby, treatment that induces repolarization of M2 macrophages to an M1 phenotype could be of benefit in tumor treatment.

Vasoactive intestinal peptide (VIP), a 26 amino acid peptide, was originally identified as a vasodilatory peptide secreted from the gastrointestinal system^10^, and nowadays is known to be released from tumors and immune cells^11–13^. VIP actions are mediated through two subtypes of G-protein coupled receptors, VPAC1 and VPAC2. VPAC receptors signal through Gs or Gq proteins, leading to an increase in cAMP and IP3 production. However, the potency to stimulate cAMP production is much higher than IP3 generation. Depending on the cell type, the activation of VPACs in immune cells mainly increases adenylyl cyclase activity and cAMP production^11–13^. VPAC receptors are expressed on immune cells. VPAC1 is constitutively expressed in T cells, mast cells and resting human monocytes/macrophages, whereas VPAC2 expression is induced by immune stimulation^14^. VIP has potent anti-inflammatory cytokine activity and downregulates pro-inflammatory responses^12,14^, and plays an important role in the regulation of the immune activity of CD4^+^ T cells by decreasing Th1 cells and increasing Th2 cells^11,12^. VIP also enhances regulatory T cell function^11,12^. In terms of macrophages, it has been demonstrated that VIP, via its receptors VPAC1 and VPAC2, activates anti-inflammatory pathways and attenuates inflammatory cytokine gene expression in both mouse and human macrophages after lipopolysaccharide (LPS) incubation^15,16^.

By modifying VIP sequences, VIPhybrid (VIPhyb) was constructed of the 22 C-terminal amino acids of VIP, which are necessary for binding to VIP receptors, and an N-terminal sequence from neurotensin (neurotensin6-11-VIP7-28)^17^. Therefore, VIPhyb maintains the ability to bind to VPACs without activating the VPAC signaling pathway^18,19^. VIPhyb has been intensively used in determining the role of VIP in immune function. Recent studies have demonstrated that inhibition of VIP signaling has a favorable outcome in viral infection and lymphoma models by enhancing CD8^+^ T cell proliferation and function^20–22^. Furthermore, VIP antagonist increased activated CD8^+^ T cell in tumor-bearing mice engrafted with pancreatic cancer cell lines^23^ However, the effect of VIP antagonist on macrophage polarization and function in tumor models remains unknown.

In the current study, we demonstrated that VIP antagonist enhanced M1 gene expression and phagocytosis of CT26 colon cancer cells in mouse macrophage RAW264.7 cells incubated with conditioned medium derived from CT26 cells. In immunodeficient SCID mice lacking T and B cells, VIP antagonist alone or in combination with anti-PD-1 reduced CT26 tumor growth, which was associated with increases in M1/M2 macrophage ratio and macrophage phagocytosis of CT26 in the tumors. Furthermore, *Vipr2* gene silencing or VPAC2 activation played an important role in regulating macrophage gene expression. These data have indicated that VIP antagonist, at least in part through VPAC2, plays a role in enhancing macrophage function against cancer. Thereby, the inhibition of VIP signaling may be a promising therapeutic target in colorectal cancer treatment.

## Results

### Conditioned medium derived from CT26 induced the expression of M2-related markers and VPAC in RAW264.7 cells

**Figure 1:**
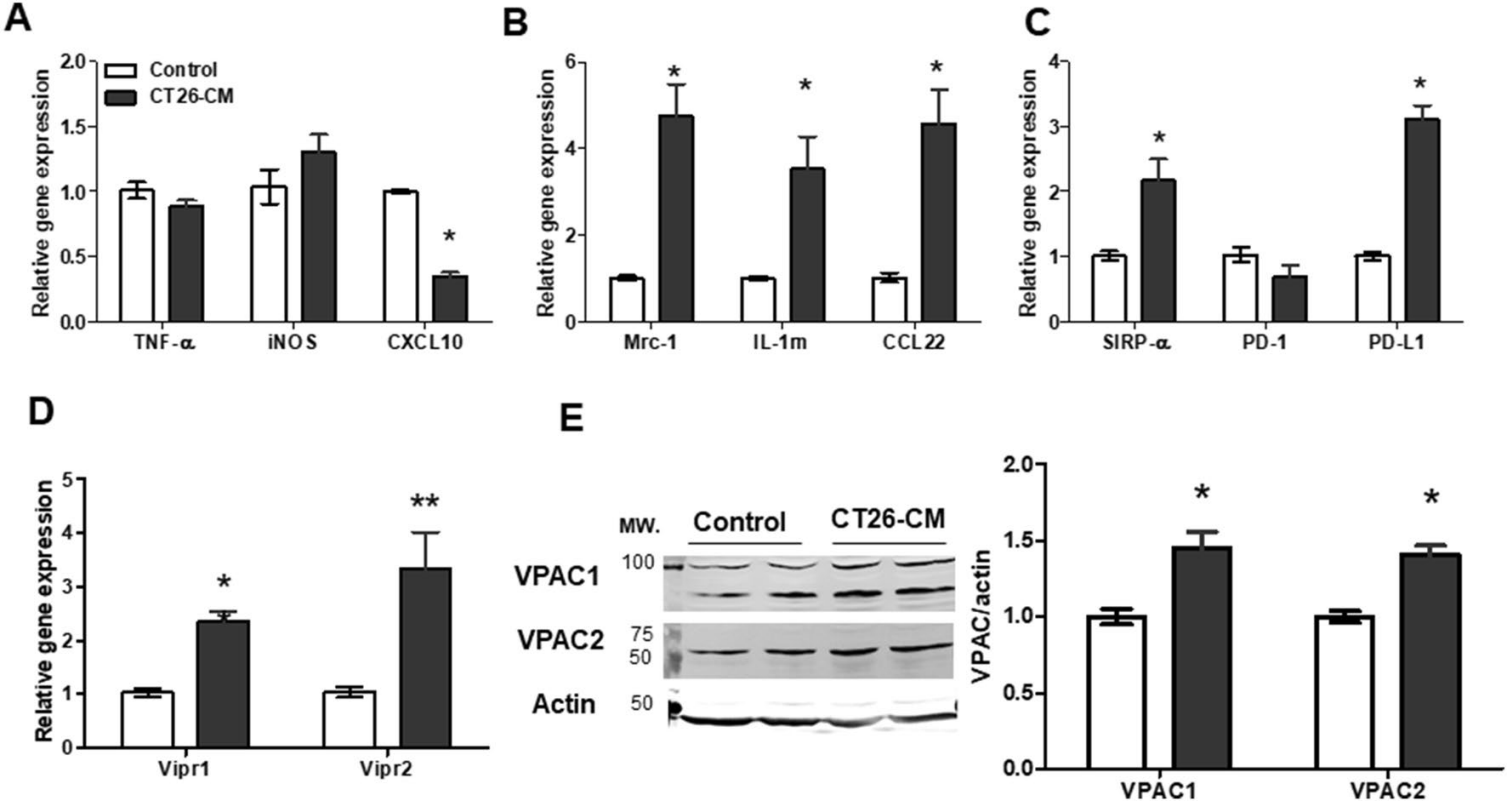
Conditioned medium from CT26 cells induced M2 macrophage polarization and VPAC expression in RAW264.7 cells. Real-time PCR analyses of M1 macrophage markers *TNF-α*, *iNOS*, and *CXCL10* (**A**), M2 macrophage markers *Mrc-1*, *IL-1rn*, and *CCL-22* (**B**), immune checkpoint markers *SIRP-α*, *PD-1*, and *PD-L1* (**C**), and VIP receptors *Vipr1* and *Vipr2* (**D**) in RAW264.7 cells at day 4 after incubation with 20% conditioned medium derived from CT26 with DMEM complete medium (CT26-CM) or 20% RPMI medium with DMEM complete medium (control); n = 5–6/group. Representative western blotting images showing protein expression levels of VPAC1 and VPAC2 in RAW264.7 cells at day 4 after incubation with CT26-CM or control (**E**, left). Western blotting analysis of VPAC1 and VPAC2 protein expression in RAW264.7 cells at day 4 after incubation with control medium or CT26-CM (E, right); n = 5/group. The full-length blots of (**E**) are included in Supplemental Fig. S1. *p < 0.05 vs control, **p < 0.01 vs control.

Tumors release numerous soluble factors into the tumor microenvironment to regulate immune function^24^. It has been shown that soluble factors released from tumors an induce M1 polarization profile from days 1–3 after incubation. In contrast, the M2 polarization profile constantly increases from days 3–5^25^. Therefore, we first examined whether tumor-derived factors found in conditioned medium collected from CT26 cells, which contains CT26 tumor-derived factors, affected macrophage polarization and VPAC expression in RAW264.7 cells. RAW264.7 cells were incubated with 20% conditioned medium derived from CT26 with DMEM complete medium (CT26-CM) or 20% RPMI medium with DMEM complete medium (control) and examine gene expression of macrophage markers and VPAC expression. As previously reported^26,27^, CT26-CM significantly reduced *CXCL10* mRNA expression and had no effect on *TNF-α* and *iNOS* mRNA expression in RAW264.7 cells (Fig. 1A). However, CT26-CM-treated RAW264.7 cells had significantly increased mRNA expression of M2-related markers mannose receptor (*Mrc1*), interleukin-1 receptor antagonist (*IL-1rn*), and *CCL-22* compared with control medium-treated cells (Fig. 1B). Because the expression of immune checkpoints such as SIRP-α and PD-1/PD-L1 affects macrophage polarization and function^28,29^, we further checked the effect of CT26-CM on immune checkpoint markers in RAW264.7 cells. We found that CT26-CM-treated RAW264.7 cells demonstrated an increase in *SIRP-α* and *PD-L1* mRNA expression compared with the controls (Fig. 1C). However, no change in *PD-1* mRNA expression was observed between the groups (Fig. 1C).

To examine whether CT26-CM could affect VPAC expression in RAW264.7 cells, we checked VPAC1 and VPAC2 expression in RAW264.7 cells after incubation with CT26-CM or control. We found that CT26-CM-treated RAW264.7 cells showed an increase in both *Vipr1* and *Vipr2* mRNA expression compared with the control-treated group (Fig. 1D). Similarly, protein expression of VPAC1 and VPAC2 was increased in CT26-CM-incubated RAW264.7 cells (Fig. 1E). These data suggest that CT26-CM induced M2 polarization and activated VPAC expression in RAW264.7 cells.

### VIP antagonist increased M1 polarization and phagocytosis of CT26 in CT26-CM-treated RAW264.7 cells

**Figure 2:**
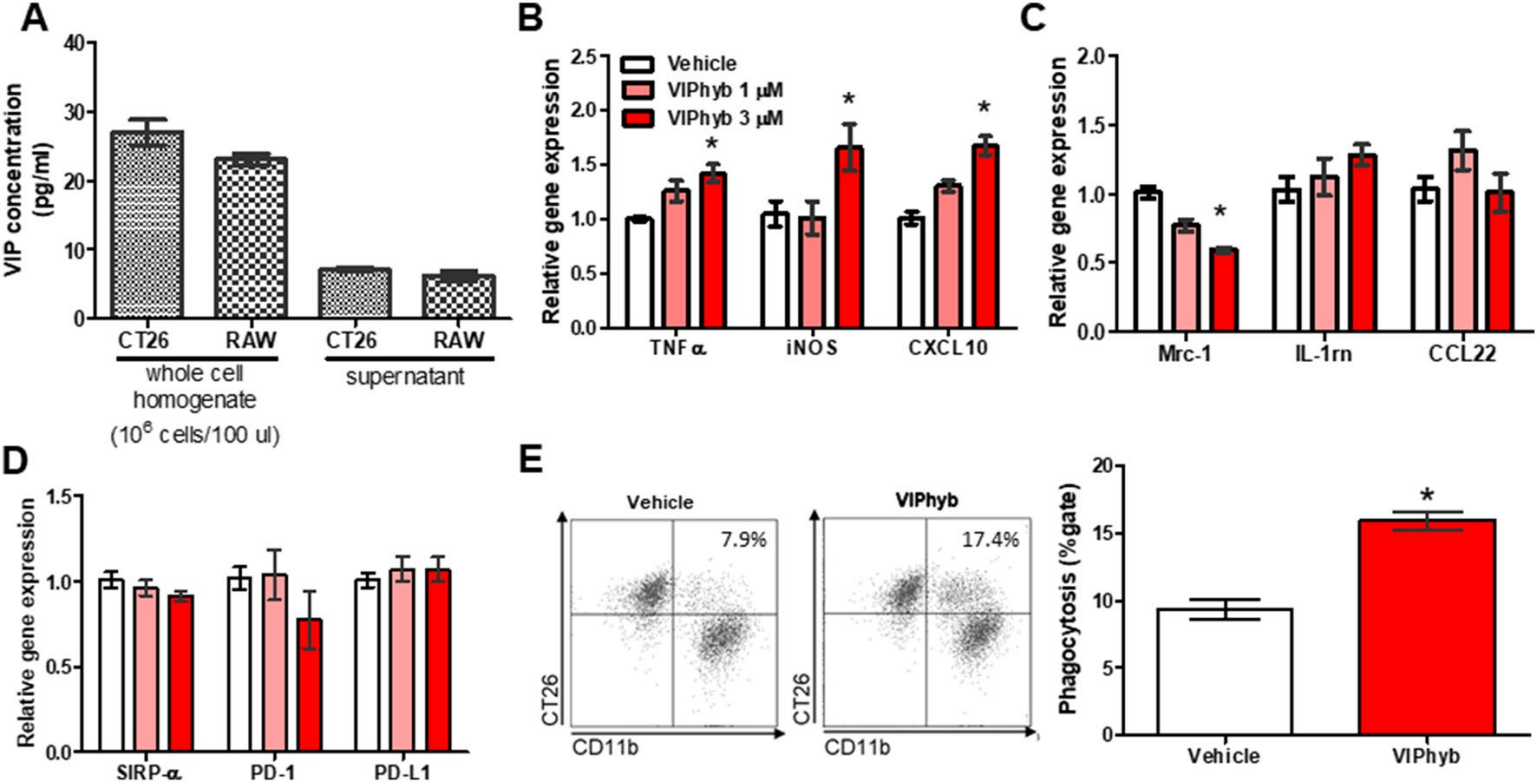
VIP antagonist promoted M1 polarization and macrophage phagocytosis of CT26 in CT26-CM-incubated RAW264.7 cells. VIP concentration in whole-cell homogenate and supernatant collected from CT26 and RAW264.7 cells (**A**); n = 4–6/group. The effect of VIP hybrid (VIPhyb, a VIP antagonist) at 1 and 3 µM on mRNA expression of M1 macrophages (**B**), M2 macrophages (**C**), and immune checkpoint (**D**) markers in RAW264.7 cells at day 4 after incubation with CT26-CM. PBS was used as vehicle; n = 7–9/group. Representative FACS histograms and flow cytometric analysis of engulfed CT26 cells of CT26-CM-incubated RAW264.7 cells treated with vehicle (PBS) or VIPhyb at 3 µM (**E**). Gating strategies for phagocytosis of PKH-26-labeled CT26 in RAW264.7 cells are shown in Supplemental Fig. S2; n = 6/group. Data from each treatment were compared with data from the control; *p < 0.05 vs vehicle.

Tumor cells and macrophages are a source of VIP secretion^11–13^. In our system, we confirmed that CT26 and RAW264.7 cells contained and secreted VIP into the supernatant (Fig. 2A). We next examined whether VIP signaling plays a role in macrophage polarization and function. We found that VIP hybrid, a competitive antagonist of VIP^17^, at a dose of 3 µM significantly enhanced *TNF-α*, *iNOS*, and *CXCL10* gene expression in CT26-CM-treated RAW264.7 cells (Fig. 2B). Conversely, VIP antagonist significantly reduced *Mrc1* gene expression but had no effect on *IL-1rn* and *CCL-22* gene expression in CT26-CM-treated RAW264.7 cells (Fig. 2C). VIP antagonist also had no effect on *SIRP-α*, *PD-1*, and *PD-L1* mRNA expression in CT26-CM-treated RAW264.7 cells compared with the vehicle-treated group (Fig. 2D). We further analyzed the effect of VIP antagonist on the phagocytic function in CT26-CM-treated RAW264.7 cells. We found that VIP antagonist enhanced the phagocytic activity against CT26 in CT26-CM-treated RAW264.7 cells (Fig. 2E). These data suggest that inhibition of VIP signaling enhances M1 macrophage polarization and macrophage phagocytic function.

### VIP antagonist reduced tumor growth and enhanced M1/M2 ratio of tumor-associated macrophages in CT26 tumor-bearing SCID mice

**Figure 3:**
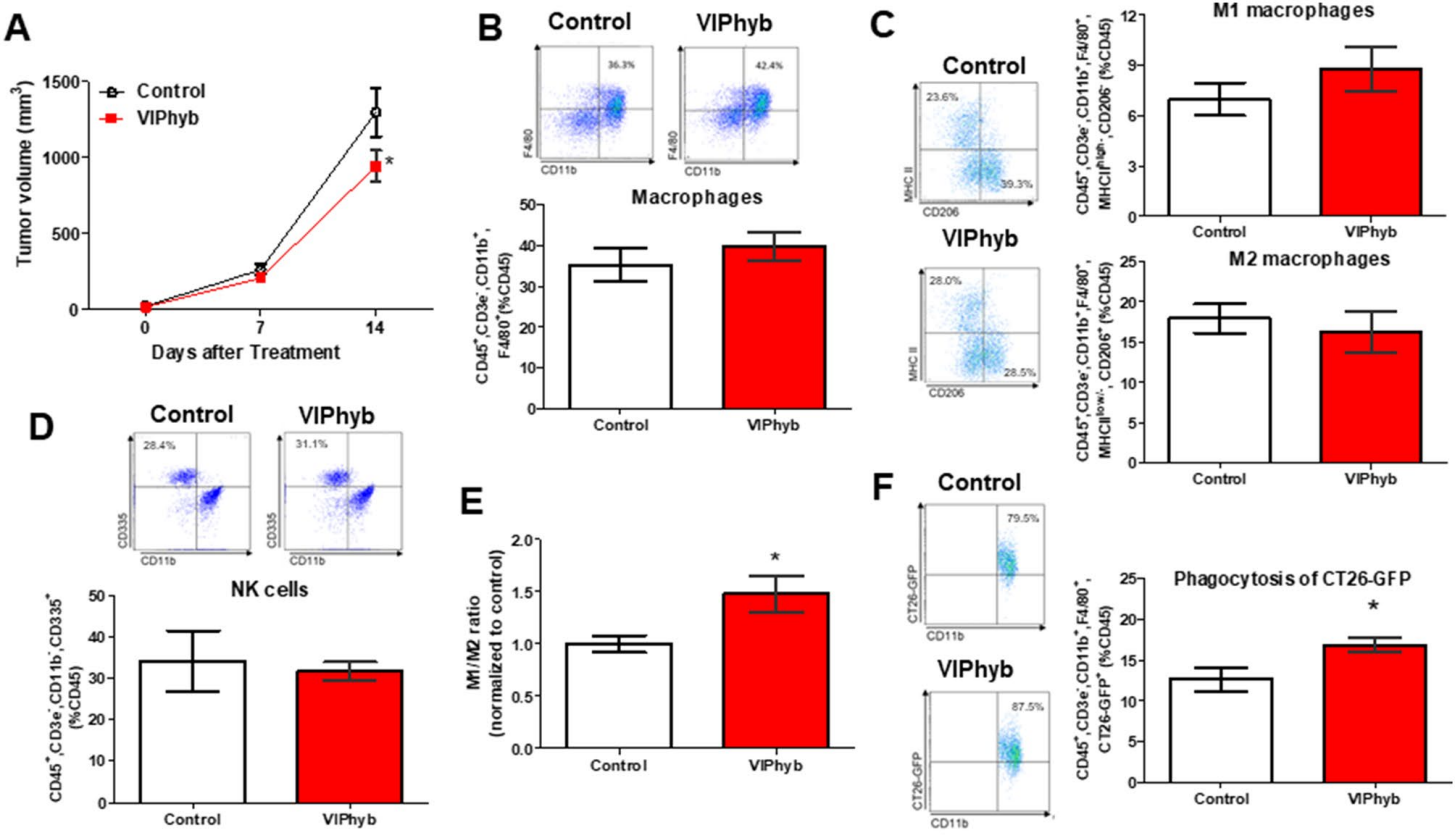
VIP antagonist significantly reduced CT26-tumor growth via enhancing macrophage phagocytosis of CT26 and M1/M2 TAM ratios in immunodeficient SCID mice. Tumor volume during 2-week treatment (**A**); n = 5–6/group. Representative FACS histograms and flow cytometric analysis of macrophages (**B**), M1 and M2 macrophages (**C**), NK cells (**D**), M1/M2 macrophage ratio (**E**), and phagocytosis of CT26-GFP (**F**) of tumor-infiltrating leukocytes isolated from PBS (control) or VIP antagonist-treated groups. Gating strategies for macrophages and NK cells isolated from tumors are shown in Supplemental Fig. S3. The value in representative FACS histograms was shown as %gate. The data in bar graphs were calculated based on %CD45. M1/M2 ratio was calculated by M1 events/M2 events. The value of the M1/M2 ratio of each treatment was then normalized to the value of the M1/M2 ratio of controls that were performed on the same day; n = 4–5/group. *p < 0.05 vs control.

To examine the effect of VIP antagonist on tumor growth and TAM function, we implanted CT26 colon cancer cells subcutaneously into immunodeficient SCID mice. After 14 days of treatment, we found that VIP antagonist significantly reduced tumor growth in SCID mice compared with the controls (Fig. 3A). The analysis of tumor-infiltrating leukocytes demonstrated that VIP antagonist did not significantly change the population of macrophages (Fig. 3B), M1 and M2 macrophages (Fig. 3C), as well as NK cells (Fig. 3D) in the implanted tumors compared with the controls. The M1/M2 macrophage ratio in the TME is correlated with patient prognosis^8,9^. For this reason, we analyzed the ratio of M1/M2 TAMs and found that the VIP antagonist-treated group had a higher M1/M2 TAM ratio than the controls (Fig. 3E). We next examined the phagocytic function of TAMs. We found that TAMs isolated from the VIP antagonist-treated group had an increase in phagocytic activity against CT26-GFP (Fig. 3F).

PD-1 expression has been reported to affect phagocytic activity in macrophages^28^. For this reason, we further determined the expression of PD-1 in isolated TAMs and found that PD-1 was slightly increased in TAMs isolated from VIP antagonist-treated mice (Supplemental Fig. S4).

### VIP antagonist in combination with anti-PD-1 antibody further reduced tumor growth in CT26 tumor-bearing SCID mice

**Figure 4:**
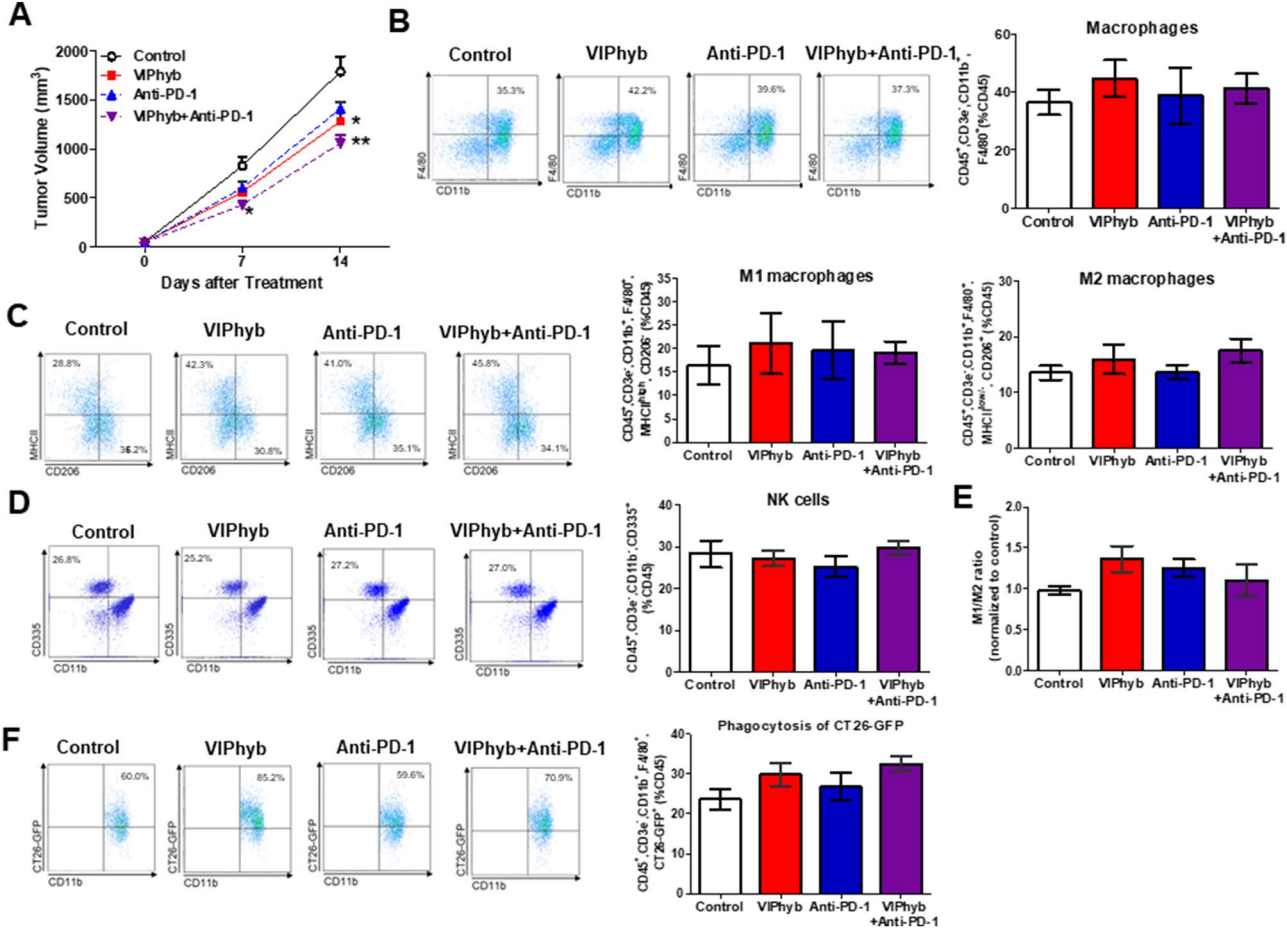
VIP antagonist in combination with anti-PD-1 antibody significantly reduced CT26-tumor growth in immunodeficient SCID mice. Tumor volume during 2-week treatment (**A**); n = 6–8/group. Representative FACS histograms and flow cytometric analysis of macrophages (**B**), M1 and M2 macrophages (**C**), NK cells (**D**), M1/M2 macrophage ratio (**E**), and phagocytosis of CT26-GFP (**F**) of tumor-infiltrating leukocytes isolated from each group. PBS was used as control. Gating strategies for macrophages and NK cells isolated from tumors are shown in Supplemental Fig. S3. The value in representative FACS histograms was shown as %gate. The data in bar graphs were calculated based on %CD45. M1/M2 ratio was calculated by M1 events/M2 events. The value of the M1/M2 ratio of each treatment was then normalized to the value of the M1/M2 ratio of controls that were performed on the same day. n = 4–5/group. Data from each treatment were compared with data from the control; *p < 0.05, **p < 0.01 compared with controls on the same day.

PD-1 tended to increase in TAMs isolated from VIP antagonist-treated mice, and therefore we hypothesized that the combined treatment of VIP antagonist and anti-PD-1 antibody may have an additional effect on reducing tumor growth and enhancing macrophage function. As previously shown, VIP antagonist significantly attenuated tumor growth after 14 days of treatment (Fig. 4A). Treatment with anti-PD-1 antibody tended to reduce tumor growth compared with the control. Interestingly, the combination of VIP antagonist and anti-PD-1 antibody significantly reduced tumor volume compared with the control and tended to further attenuate tumor growth compared with the VIP antagonist or anti-PD-1 antibody alone (Fig. 4A).

Analysis of tumor-infiltrating leukocytes revealed no differences in macrophages (Fig. 4B), M1 and M2 macrophages (Fig. 4C), NK cell population (Fig. 4D) in implanted tumors among the groups. VIP antagonist showed a tendency of enhanced M1/M2 macrophage ratios (Fig. 4E). VIP antagonist alone or in combination therapy with anti-PD-1 antibody tended to increase macrophage phagocytosis of CT26-GFP compared with the controls (Fig. 4F).

### Disruption of VPAC2 signaling affected macrophage polarization in CT26-CM-treated RAW264.7 cells

**Figure 5:**
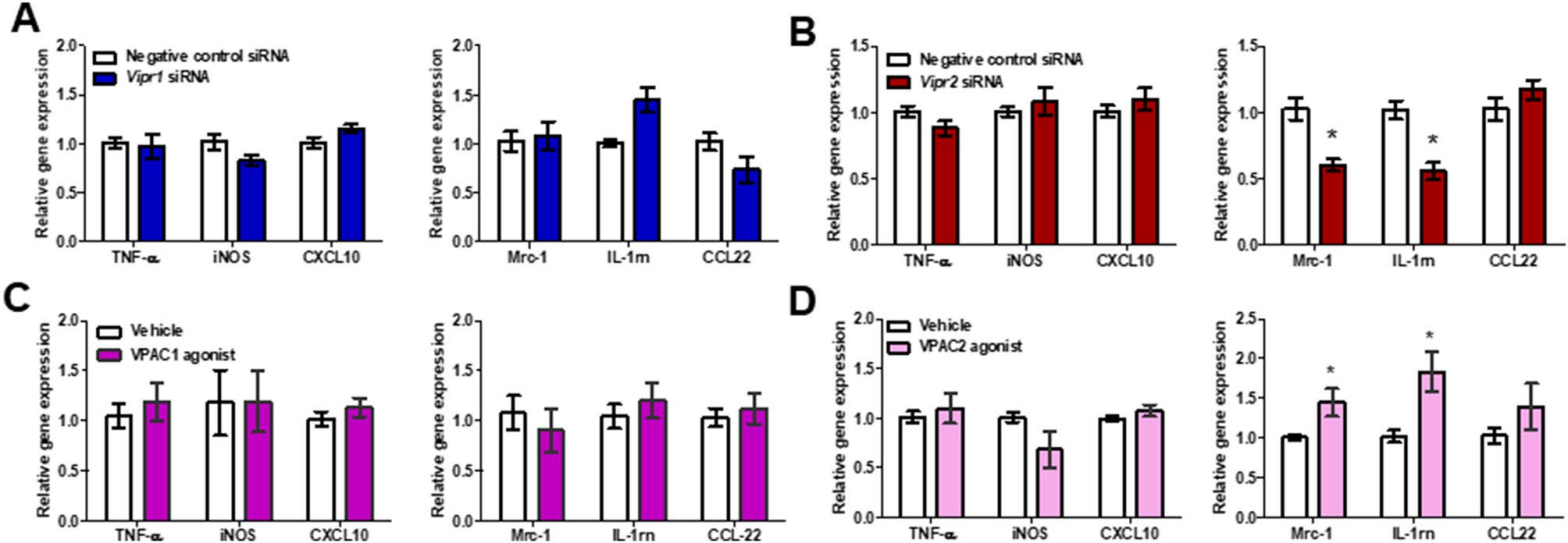
Knockdown or activation of VPAC2 disturbed macrophage polarization in RAW264.7 cells incubated with CT26-CM. Effect of *Vipr1* (**A**) or *Virp2* (**B**) silencing on gene expression of M1 and M2 macrophage markers in RAW264.7 cells at day 4 after incubation with CT26-CM. Negative control siRNA was used as control; n = 6–9/group. Effect of [Lys15, Arg16, Leu27] VIP (1–7)/GRF (8–27), a VPAC1 agonist, (**C**) or BAY 55–9837, a VPAC2 agonist, (**D**) on the mRNA expression of M1 and M2 macrophage markers in RAW264.7 cells at day 3 after incubation with CT26-CM. PBS was used as vehicle; n = 6–10/group. *p < 0.05 vs Negative control siRNA or vehicle.

Next, we wanted to clarify which VPAC subtype was responsible for CT26-CM-induced M2 polarization in RAW264.7 cells. We transfected *Vipr1* or *Vipr2* siRNA into CT26-CM-incubated RAW264.7 cells. Compared with the negative control siRNA, either *Vipr1* (Supplemental Fig. S5A) or *Vipr2* (Supplemental Fig. S5B) siRNA knockdown resulted in a 25–30% reduction in *Vipr1* or *Vipr2* mRNA expression, respectively. *Vipr1* siRNA did not change either M1 or M2 macrophage markers compared with the negative control siRNA (Fig. 5A). However, *Vipr2* silencing resulted in a significant reduction of *Mrc-1* and *IL-1rn* mRNA expression but had no effect on M1 macrophage markers in CT26-CM-incubated RAW264.7 cells (Fig. 5B).

We further confirmed the role of VPAC2-induced M2 macrophage polarization in CT26-CM-incubated RAW264.7 cells using [Lys15, Arg16, Leu27] VIP (1–7)/GRF (8–27), a selective VPAC1 agonist^30^, or BAY 55-9837, a selective VPAC2 agonist^31^. We found that VPAC1 agonist did not affect either M1 or M2 macrophage mRNA expression (Fig. 5C). In contrast, VPAC2 agonist significantly increased *Mrc1* and *IL-1rn* but not *CCL-22* gene expression in CT26-CM-incubated RAW264.7 cells (Fig. 5D). VPAC2 agonist showed a tendency to reduce *iNOS* mRNA expression; however, it did not affect *TNF-α* and *CXCL10* gene expression (Fig. 5D).

### Expression of VPAC in tumor-associated macrophages in human colorectal cancer tissues

**Figure 6:**
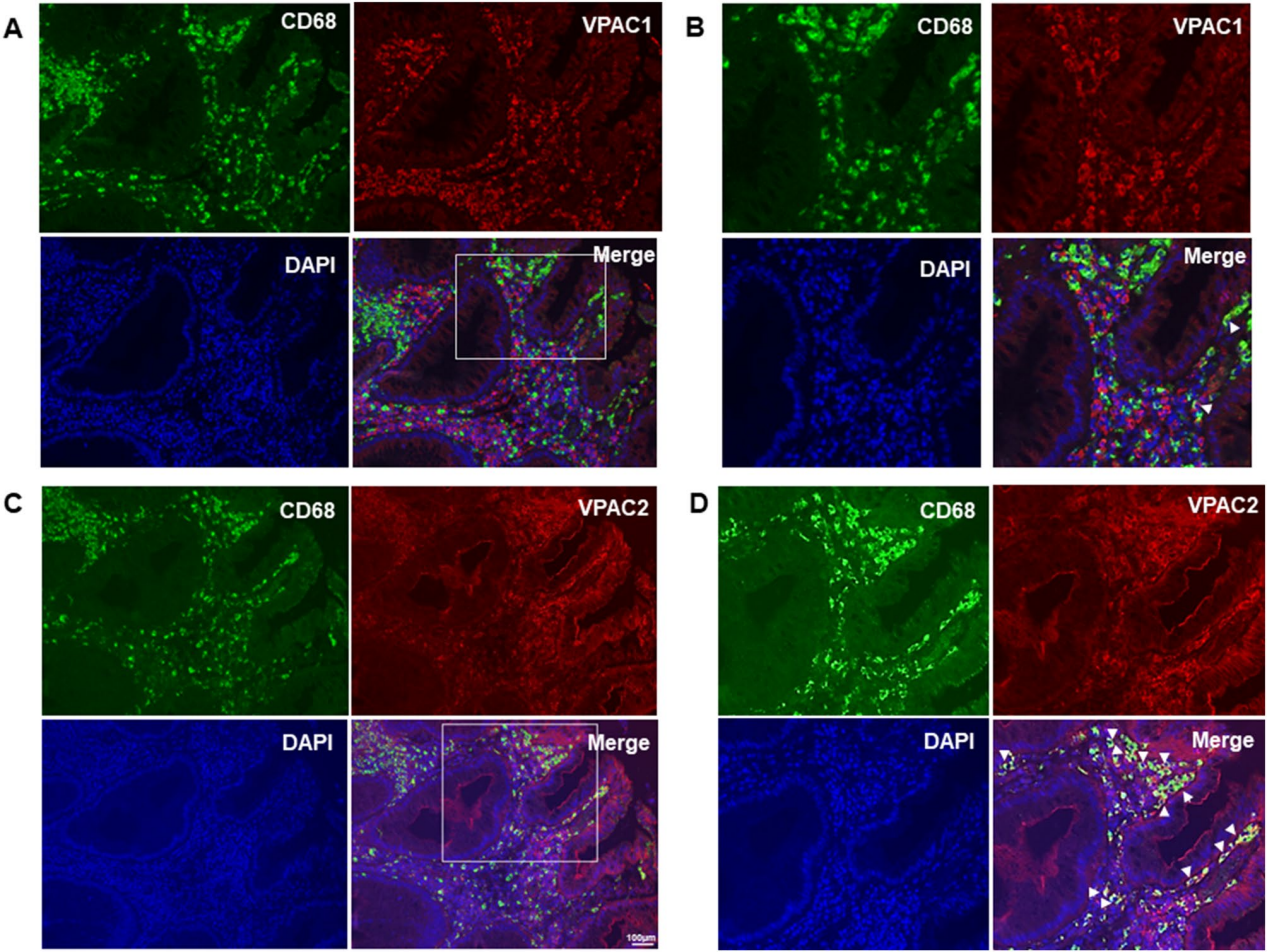
CD68 macrophages were highly expressed in tumor stroma and co-expressed with VPAC2, but not VPAC1, in human colorectal cancer specimens. Representative images of CD68 (green) and VPAC1 (red; **A**,**B**), and CD68 (green) and VPAC2 (red; **C**,**D**) staining in the tumor stroma of human colorectal cancer specimens. B and D were enlarged images from white square region indicated in (**A**) and (**C**), respectively. All images represented cancer lesions. Nuclei were stained by DAPI (blue). White arrow indicated double positive cells. Scale bar 100 µm.

Our data revealed the role of VPAC, mainly via VPAC2, in macrophage polarization and function in mice. Therefore, we examined the distribution of VPAC in human colorectal cancer tissues. The expression of CD68—a macrophage marker—was highly expressed in the tumor stroma (Fig. 6); however, a lesser degree of CD68-positive cells was identified in adjacent normal tissue (Supplemental Fig. S6). In contrast, both VPAC1 and VPAC2 expression was found in cells located in the tumor stroma (Fig. 6) and the interstitial compartment of adjacent normal tissue (Supplemental Fig. S6). In tumor stroma, CD68-expressing cells were not positive for VPAC1 staining (Fig. 6A,B). In contrast, co-localization with the macrophage marker CD68 was observed in VPAC2-positive cells (Fig. 6C,D).

## Discussion

VIP expression has been shown to correlate with advanced tumor stage in colorectal carcinoma^13,32^. In the immune system, VIP has a potent effect on immunomodulators and plays a critical role in promoting anti-inflammatory immune effects^12^. In the current study, we demonstrated that VIP antagonist enhanced M1 markers *TNF-α*, *iNOS*, and *CXCL10*, reduced *Mrc-1* mRNA expression, and increased phagocytic ability against CT26 colon cancer cells in mouse macrophage RAW264.7 cells incubated with CT26-CM. Furthermore, VIP antagonist alone or in combination with anti-PD-1 antibody significantly reduced tumor size in CT26 tumor-bearing SCID mice, at least in part, via an increase in M1/M2 macrophage ratio and TAM phagocytic function. Finally, our data indicated that these beneficial effects of VIP antagonist were likely mediated through the inhibition of VPAC2.

In the current study, we demonstrated that inhibition of both VPACs by VIP antagonist promoted M1 polarization, with reduced *Mrc1* expression in RAW264.7 cells incubated with CT26-CM. We further investigated the role of each VPAC in macrophage polarization. We found that the disruption of VPAC2 signaling, but not VPAC1 signaling, by agonist or siRNA targeting of each VPAC subtype affected only M2 polarization and not M1 polarization. These discrepancy effects between inhibiting both VPACs by VIPhyb and using siRNAs against each subtype may be caused by the insufficient reduction of VPAC by siRNA (knockdown efficiency was 25%–30%). Further experiments need to be performed to confirm the effect of each VPAC, especially VPAC2, on macrophage polarization and function using a specific antagonist of each VPAC subtype.

In SCID mice, VIP antagonist alone significantly reduced tumor growth. Phagocytic ability and M1/M2 TAM ratio were increased in the VIPhyb group with no differences in M1 and M2 infiltration at the tumor site (Fig. 3). We further confirmed that VIPhyb alone or in combination with anti-PD-1 antibody reduced tumor growth compared with the control. However, M1/M2 TAM ratio and phagocytosis ability tended to increase in the VIPhyb-treated group compared with the controls (Fig. 4). The differences in the effect of VIPhyb on the M1/M2 TAM ratio and the phagocytosis of CT26-GFP between the two figures may be explained by statistical analysis. Figure 3 was analyzed by Student’s t-test, while Fig. 4 was analyzed by one-way ANOVA.

Generally, VPAC1 is constitutively expressed in resting human monocytes/macrophages, whereas VPAC2 expression is induced by immune stimulation^14^. In inflammatory diseases, it has been reported that high VPAC1 expression in macrophages is correlated with chronic pro-inflammatory environments. Alveolar macrophages from COPD patients have upregulated VPAC1 and VPAC2 expression, which is more prominent for VPAC1 than for VPAC2^33^. In the same way, CD163^+^ macrophages from the synovial fluid of patients with rheumatoid arthritis expresses both VPAC1 and VPAC2 at higher levels than macrophages from non-inflamed synovium^15^. However, little is known about VPAC expression on TAMs in human cancer tissue. Our results revealed that TAMs preferentially expressed VPAC2 in human colorectal cancer, while very little VPAC1 and VPAC2 protein was expressed on macrophages in the adjacent normal tissues. The degree of inflammation at the site of the disease might determine the pattern of VPAC expression. Tumor cells release chemokines and cytokines to create an anti-inflammatory state in the TME to support their growth^34^. Thereby, such an environment may induce upregulation of VPAC2, but not VPAC1, in human macrophages.

Studies have demonstrated that VIP plays a crucial role in promoting M2 macrophage polarization in inflammatory environments induced by LPS and/or IFNγ^11,15,35,36^. Similarly, VPAC2 agonist induced M2-related gene markers but reduced M1-related gene markers in macrophages derived from human monocytes by GM-CSF (GM-MØ) stimulated with LPS. In contrast, VPAC1 agonist showed prominent effects in reducing M1 macrophage gene expression, without significant changes in M2 macrophage gene expression in GM-MØ^15^. Similarly, Dewit et al. showed that VPAC2 agonist had inhibitory effects on LPS-induced TNFα and IL-12 synthesis without affecting IL-10 production in macrophages^37^. These data suggested that activation of VIP signaling inhibited pro-inflammatory cytokine production in response to inflammatory stimuli. However, little is known about the effect of VIP and its receptors on TAMs, which contain M2-like phenotypes. Chen et al. demonstrated that VIP inhibited M1-macrophage gene expression in both IL-4-/IL-13-stimulated macrophages and TAMs co-cultured with gastric cancer cells^38^. Using a VIP antagonist, we found that the inhibition of VIP induced M1 macrophage gene expression and reduced *Mrc-1*, an M2 macrophage marker, in CT26-CM-incubated mouse macrophages. Furthermore, silencing of VPAC2, but not VPAC1, or VPAC2 agonist affected M2 macrophage polarization. These data imply that the disruption of VIP and its receptors, especially VPAC2, may also play a critical role in macrophage polarization in response to anti-inflammatory environments, such as the TME.

Immune checkpoints are crucial regulators controlling immune activation against cancer cells. Several markers of immune checkpoints and their ligands, such as SIRP-α and PD-L1, have been reported to be expressed on macrophage cells and influence macrophage function. Recent studies also demonstrate that the expression and activity of immune checkpoints may affect macrophage polarization. Lin et al. demonstrated that overexpression of SIRP-α enhances M2 polarization, while deletion of SIRP-α promotes M1 polarization in bone marrow macrophages treated with LPS and IFN-γ^29^. Similarly, Wei et al. showed that an increase in PD-L1 function on macrophages leads to M2 polarization but has no effect on M1 polarization^39^. In the current study, we found that CT26-CM induced the expression of common M2-related genes *Mrc-1*, *IL-1rn*, and *CCL-22*, together with an increase in *SIRP-α* and *PD-L1* mRNA expression in RAW264.7 cells. Our results have also confirmed the possibility that SIRP-α and PD-L1 may be useful as markers for M2-like TAMs.

Pharmacological inhibition of VIP signaling has shown beneficial effects in increasing proliferation and reducing PD-1 expression on CD8^+^ and NK T cells in viral infection and lymphoma^20,22,40^. Moreover, VIP antagonist has been shown to enhance chimeric antigen receptor (CAR)-T cell therapy^41^. While CAR-T cell therapy has shown a clinical efficacy for hematological malignancies, its role in solid tumors remains controversial^42,43^. This is partly because solid tumors have high complexity in the TME, leading to the difficulty of T cells in penetrating and surviving in their environments^42,44^. Macrophages are a major population of immune cells in solid tumors and play a critical role in regulating tumor growth^4,44^. Thereby, several strategies, such as nanoparticles packed with drugs/contents or CAR-macrophages, have been proposed to preferentially change M2-like TAMs to M1-like TAMs to enhance phagocytic activity and antigen presentation of tumors^6,43,45,46^. In this study, we have further demonstrated the beneficial effect of VIP antagonist on enhancing M1/M2 TAM ratios and macrophage phagocytic function in a colon cancer model. For this reason, nanoparticles carrying VIP antagonist or genetic modification of VPAC-targeting macrophages might be a novel strategy to enhance the effectiveness and specificity of therapy against solid cancer.

In conclusion, the present study demonstrated that inhibition of VIP signaling promoted M1 polarization and increased the phagocytic function of TAMs, contributing to a reduction in tumor growth in a colon cancer mouse model. Furthermore, these effects may be in part regulated via VPAC2. Therefore, the inhibition of VIP effects, particularly via VPAC2 in macrophages, may be a promising therapeutic target to treat colorectal cancer.

## Materials and methods

### Animals

All experimental procedures were performed in accordance with the guidelines for the care and use of animals established by Kagawa University following Animal Research: Reporting In Vivo Experiments (ARRIVE) guidelines. Five-week-old male immunodeficient SCID mice (CLEA, Tokyo, Japan) were housed in animal facilities in a 12-h light–dark cycle with free access to standard chow and water.

### Cell lines and reagents

CT26 colon cancer cells (ATCC, Gaithersburg, MD) were cultured in RPMI (Wako, Osaka, Japan) supplemented with 10% FBS, 100 IU/mL penicillin, and 100 IU/mL streptomycin. To generate GFP-tagged CT26 cells (CT26-GFP), pEGFP-C1 plasmid (Clontech, Mountain View, CA) was transfected into CT26 cells using Lipofectamine 3000 (Thermo Fisher Scientific, Rockville, MD) according to the manufacturer’s instructions. CT26-GFP cells were then selected by cell sorting (Beckman Counter, Sykesville, MD) and cultured in RPMI containing 600 µg/mL geneticin (Thermo Fisher Scientific), 10% FBS, 100 IU/mL penicillin, and 100 IU/mL streptomycin. RAW264.7 cells were cultured in DMEM (Sigma–Aldrich, St. Louis, MO) with 10% FBS, 100 IU/mL penicillin, and 100 IU/mL streptomycin (DMEM complete medium).

### Preparation and collection of conditioned medium derived from CT26

CT26 cells were seeded at a density of 4 × 10^6^ cells and cultured in 20 mL RPMI medium containing 10% FBS, 100 IU/mL penicillin, and 100 IU/mL streptomycin for 3 days. Supernatant was collected and centrifuged at 400×*g* for 5 min. Then, supernatant was aliquoted and kept at − 80 °C until use.

### CT26-CM-incubated RAW264.7 cells and treatments

RAW264.7 cells (2 × 10^4^) were incubated with 20% conditioned medium derived from CT26 with DMEM complete medium (CT26-CM) or 20% RPMI medium with DMEM complete medium (control). VIP hybrid (KPRRPYTDNYTRLRKQMAVKKYLNSILN-amide, Phoenix Pharmaceutical, Burlingame, CA)—a competitive antagonist of VIP^17^—at 1 and 3 µM was added daily. In a separate experiment, [Lys15, Arg16, Leu27] VIP (1–7)/GRF (8–27) (Phoenix Pharmaceuticals)—a selective VPAC1 agonist^30^—or BAY 55-9837 (Tocris Bioscience, Minneapolis, MN)—a selective VPAC2 agonist—was added daily at a dose of 100 nM^31^. mRNA extraction was performed on day 3 or day 4 after CT26-CM incubation, depending on the experiment. PBS was used as vehicle treatment.

*Vipr1*, *Vipr2* or negative control siRNA (Thermo Fisher Scientific) was transfected into RAW264.7 cells at day 1 after CT26-CM incubation using Lipofectamine RNAimax (Thermo Fisher Scientific) following the manufacturer’s instructions. *Vipr1* or *Vipr2* mRNA expression was analyzed to evaluate knock-down efficiency at day 3 after CT26-CM incubation. M1 and M2 gene expression was measured at day 4 after CT26-CM incubation.

### Real-time PCR

RNA was extracted from RAW264.7 cells using ISOGEN (Nippon Gene, Tokyo, Japan) following the manufacturer’s instructions. cDNA was prepared and used to analyze the gene expression of M1 markers *TNF-α*, *iNOS*, and *CXCL10*, M2 markers *Mrc-1*, *IL-1rn*, and *CCL22*, VIP receptor subtypes *Vipr1* and *Vipr2*, immune checkpoint markers *SIRP-α*, *PD-1*, and *PD-L1*, and *Actb* by real-time PCR using a 7300 Fast Real-Time PCR System (Applied Biosystems, Thermo Fisher Scientific) and Light Cycler Fast Start DNA Master kit (Applied Biosystems). Relative mRNA expression levels were determined using the 2 − ΔΔCt method. The ΔΔCt value was calculated using control group data. Primer sequences are shown in Table 1.

**Table 1:** List of primers and primer sequences in real time-PCR assays.

Primer name	Forward primer	Reverse primer
TNF-α	AGCCTGTAGCCCACGTCGTA	TGGCACCACTAGTTGGTTGTCT
iNOS	CACCTTGGAGTTCACCCAGT	ACCACTCGTACTTGGGATGC
CXCL10	ATCATCCCTGCGAGCCTATCCT	GACCTTTTTTGGCTAAACGCTTTC
Mrc-1	GTTCACCTGGAGTGATGGTTCTC	AGGACATGCCAGGGTCACCTTT
IL-1rn	TGTGCCTGTCTTGTGCCAAGTC	GCCTTTCTCAGAGCGGATGAAG
CCL-22	GTGGAAGACAGTATCTGCTGCC	AGGCTTGCGGCAGGATTTTGAG
SIRP-α	TCATCTGCGAGGTAGCCCACAT	ACTGTTGGGTGACCTTCACGGT
PD-1 (CD279)	CGGTTTCAAGGCATGGTCATTGG	TCAGAGTGTCGTCCTTGCTTCC
PD-L1	GACCAGCTTTTGAAGGGAAATG	CTGGTTGATTTTGCGGTATGG
Vipr1	CGAAACTACATCCACATGCATCTC	CCATATCCTTGATGAAGACGG
Vipr2	TGCCTCTTCAGGAAGCTGCACT	TGGAGTAGAGCACGCTGTCCTT

### Phagocytic ability

On day 4 after CT26-CM incubation, RAW264.7 cells were co-incubated with PKH-26-labeled CT26 (Sigma–Aldrich) in accordance with the manufacturer's instructions at a ratio of 1:1 for 2 h. Then, CD11b-FITC or isotype control for CD11b staining (eBioscience, Thermo Fisher Scientific) was used to stain RAW264.7 cells at 4 °C for 20 min in PBS with FASC buffer, and then washed twice. Phagocytic ability was analyzed with a CytoFLEX (Beckman Coulter) using CytExpert Software (Beckman Coulter). Gating strategies for phagocytosis of PKH-26-labeled CT26 in RAW264.7 cells are shown in Supplemental Fig. S2.

### Tumor implantations and treatments

CT26-GFP (1 × 10^5^) were implanted subcutaneously (s.c.) into 6-week-old SCID mice. When tumors were established, mice were received either PBS or VIP hybrid (10 µg/day, s.c., daily; KareBay Biochem, Monmouth Junction, NJ) for 2 weeks. For the combination study with anti-PD-1 antibody, mice were divided into four groups: (1) PBS; (2) VIP hybrid (10 µg/day, s.c., daily); (3) anti-PD-1 antibody (200 µg/day, intraperitoneal, 3 times/week; Bio X cell, Lebanon, NH): or (4) combined therapy of VIP hybrid and anti-PD-1 antibody for 2 weeks. Tumor size was measured by caliber every week. Tumor volume was calculated by length*(width*width)/2.

### Preparation of single cell suspensions from tumors

In a separate group of mice, tumors were removed after 12–14 days of each treatment and mechanically dissociated with surgical scissors and digested with collagenase IV (Sigma–Aldrich), dispase II (Sigma–Aldrich), and DNase I (Thermo Fisher Scientific) in PBS for 20 min at 37 °C with shaking. After enzymatic dissociation, the samples were transferred to ice to stop the reaction. The tumor suspension was then strained using 100- and 70-μm cell strainers (Thermo Fisher Scientific) and washed with FACS buffer (5% FBS, 0.1% sodium azide in PBS) and centrifuged at 400×*g* at 4 °C. Leukocytes were further separated from contaminating tumor cells by gradient centrifugation using Histopaque-1083 (Sigma–Aldrich) at 400×*g* for 30 min at room temperature. Enriched leukocytes were collected, washed, and resuspended with FACS buffer in separated tubes. Samples were kept on ice until use.

### Flow cytometry

Tumor-infiltrating leukocytes were blocked with 1 μg/10^6^ cells CD16/CD32 (eBioscience, Thermo Fisher Scientific) for 10 min on ice, before staining with a series of antibodies. Antibody information is listed in Table 2. The optimal concentration of each antibody was determined prior to each experiment. Dead cells were stained with a LIVE/DEAD fixable near-IR dead cell stain kit (Thermo Fisher Scientific). Leukocyte populations were defined as follows: M1 macrophages (CD45^+^, CD3e^−^, CD11b^+^, F4/80^+^, MHCII^high^, CD206^-^), M2 macrophages (CD45^+^, CD3e^−^, CD11b^+^, F4/80^+^, MHCII^low/-^, CD206^+^), natural killer (NK) cells (CD45^+^, CD3e^−^, CD11b^+^, CD335^+^), macrophage phagocytosis of CT26 cells (CD45^+^, CD3e^−^, CD11b^+^, F4/80^+^, CT26-GFP^+^), and PD-1^+^ macrophages (CD45^+^, CD3e^−^, CD11b^+^, F4/80^+^, PD-1^+^). Cell suspensions were stained with relevant antibodies at 4 °C for 20 min in FASC buffer, washed twice, and analyzed with a CytoFLEX (Beckman Coulter) using CytExpert Software (Beckman Coulter). Gates were set using negative controls to determine the level of true-positive staining. Gating strategies for macrophages and NK cells isolated from tumors are shown in Supplemental Fig. S3. M1/M2 ratio was calculated by M1 events/M2 events. The value of the M1/M2 ratio of each treatment was then normalized to the value of the M1/M2 ratio of controls that were performed on the same day.

**Table 2:** List of antibodies and their application in the present study.

Antibody	Use	Clone	Company
Mouse CD45	FACS	30-F11	eBioscience
Mouse CD3e	FACS	145-2C11	eBioscience
Mouse CD11b	FACS	M1/70	eBioscience
Mosue F4/80	FACS	BM8	eBioscience
Mosue MHCII	FACS	M5/114.15.2	eBioscience
Mouse CD206	FACS	C068C2	Biolegend
Mouse CD335	FACS	2971.4	eBioscience
Mouse PD-1	FACS	J43	eBioscience
Mouse PD-1	Treatment	29F.1A12	Bio X cell
Mouse VPAC2	Western blotting	5B3	Santa Cruz
Mouse β-actin	Western blotting	AC-15	Sigma-aldrich
Human/mouse VPAC1	Western blotting	SP234	Abcam
Human VPAC2	Western blotting	SP235	Abcam
Human CD68	Western blotting	PG-M1	Dako

### Western blotting analysis

CT26-CM-incubated RAW264.7 cells or controls were lysed using RIPA buffer (Thermo Fisher Scientific) following the manufacturer's instructions, and lysates were centrifuged at 12,000 rpm for 10 min at 4 °C. A total of 75 μg protein was loaded and separated by 12% sodium dodecyl sulfate polyacrylamide gel, and transferred to nitrocellulose filter membranes. Then, the membranes were blocked in Blocking Buffer (LI-COR, Lincoln, NE,) at room temperature for 1 h and probed by VPAC1 (1:200) or VPAC2 (1:200) at 4 °C overnight. The blots were washed and incubated with IR-Dye800-conjugated anti-rabbit or anti-mouse secondary antibody (LI-COR) for 1 h at room temperature. The membranes were visualized by the Odyssey infrared imaging system (LI-COR). β-actin was used as an internal control.

### Patient samples and tissue processing

As described previously^47^, we obtained a series of colorectal cancer specimens from patients diagnosed with colorectal cancer by histopathological examination who underwent surgical resection at Kagawa University. Their tissue sections were kept at Kagawa University Hospital. All patients or their guardians gave their informed consent for inclusion before specimen collection. The study was conducted in accordance with the Declaration of Helsinki, and the protocol was approved by the Ethics Committee of Kagawa University.

### Immunofluorescence

Colorectal cancer tissues were fixed in 10% formalin and embedded in paraffin. These tissues were deparaffinized and blocked with 10% goat-anti serum for 1 h at room temperature. Then, the sections were incubated with antibodies against CD68 (1:100), VPAC1 (1:100), or VPAC2 (1:100) overnight at 4 °C. The tissues were washed and incubated with Alexa Fluor 488 goat anti-mouse IgG (1:500; Thermo Fisher Scientific) or Alexa Fluor 594 goat anti-rabbit IgG (1:500; Thermo Fisher Scientific) secondary antibodies for 1 h at room temperature in the dark. Nuclei were stained with 4′,6-diamidino-2-phenylindole (DAPI) for 10 min. Images were captured with a fluorescence microscope (BZ9000; KEYENCE, Itasca, IL).

### Data and statistical analyses

Results are presented as mean ± SEM. Student's t-test was used to compare data from two individual groups. Multiple group comparisons were made using one-way or two-way analysis of variance (ANOVA), followed by Tukey's multiple comparison test. All statistical analyses were performed using GraphPad Prism 6 (GraphPad Software Inc., La Jolla, CA). P-values of < 0.05 were considered statistically significant.

### Ethics declarations

This study was conducted according to the guidelines of the Declaration of Helsinki. All protocols were approved by the Institutional Review Board of Kagawa University. Experiments with animals were performed in accordance to the guidelines for experimental animal managements established by Kagawa University (protocol code 20610-2).

### Informed consent statement

Informed consents were obtained from all the patients or their guardians.

## Supplementary Information

Supplementary Information.

## Data Availability

The data used to support the findings of this study are available from the corresponding author upon request.
