# Supplementary material for: Vasoactive intestinal peptide blockade suppresses tumor growth by regulating macrophage polarization and function in CT26 tumor-bearing mice
Source: Sci Rep. 2023 Jan 17;13:927. doi: 10.1038/s41598-023-28073-6 (PMC9845384; doi:10.1038/s41598-023-28073-6)
Supplement: Supplementary file 1 — Supplementary Information. [file 41598_2023_28073_MOESM1_ESM.pdf]

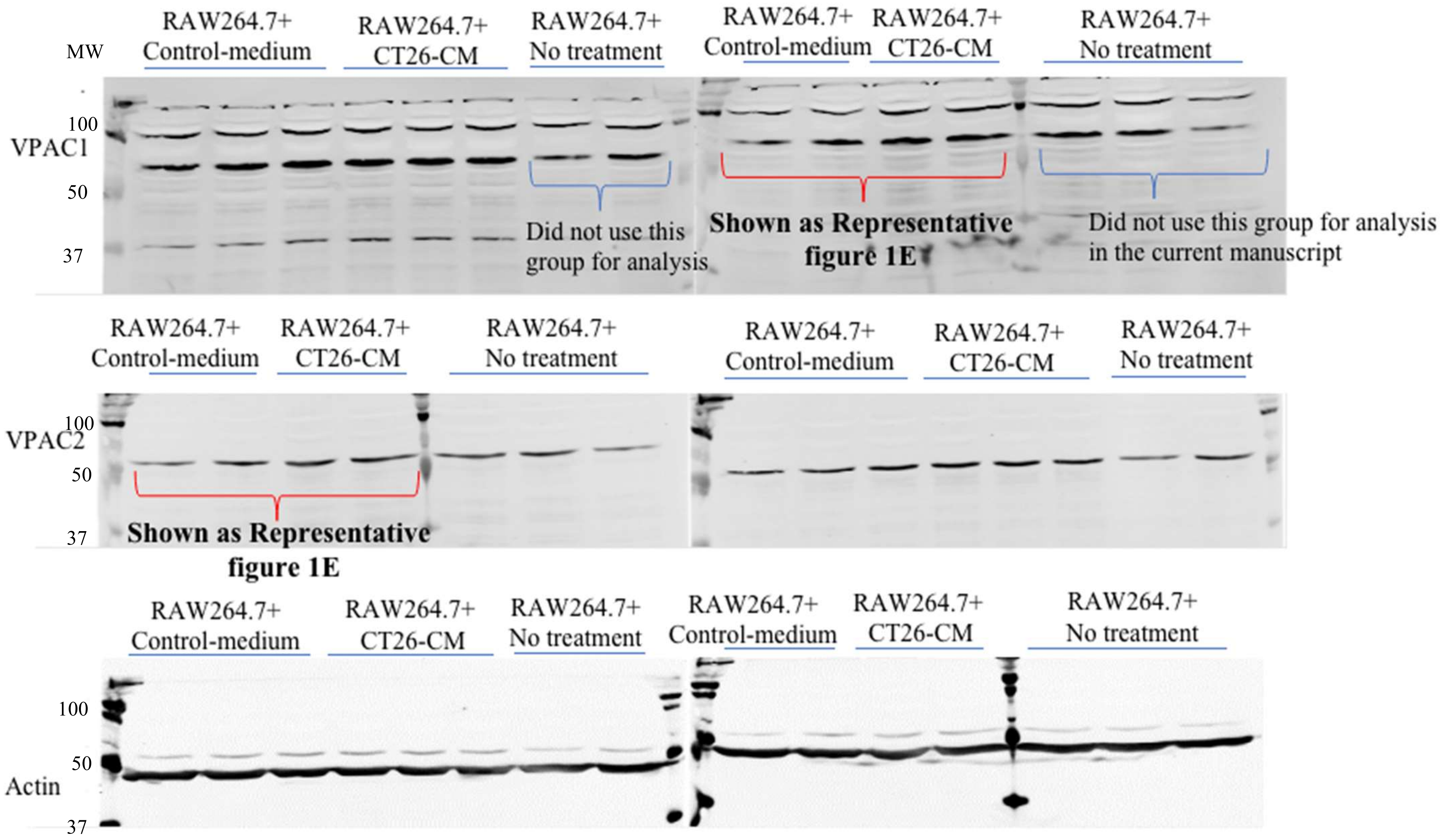

Supplemental Fig. 1: The full-length blots showing protein expression levels of VPAC1 and VPAC2 in RAW264.7 cells at day 4 after incubation with 20% conditioned medium derived from CT26 with DMEM complete medium (CT26-CM) or 20% RPMI medium with DMEM complete medium (Control-medium). RAW264.7+No treatment group was not used for analysis. RAW264.7+No treatment group did not used for analysis in the current manuscript.

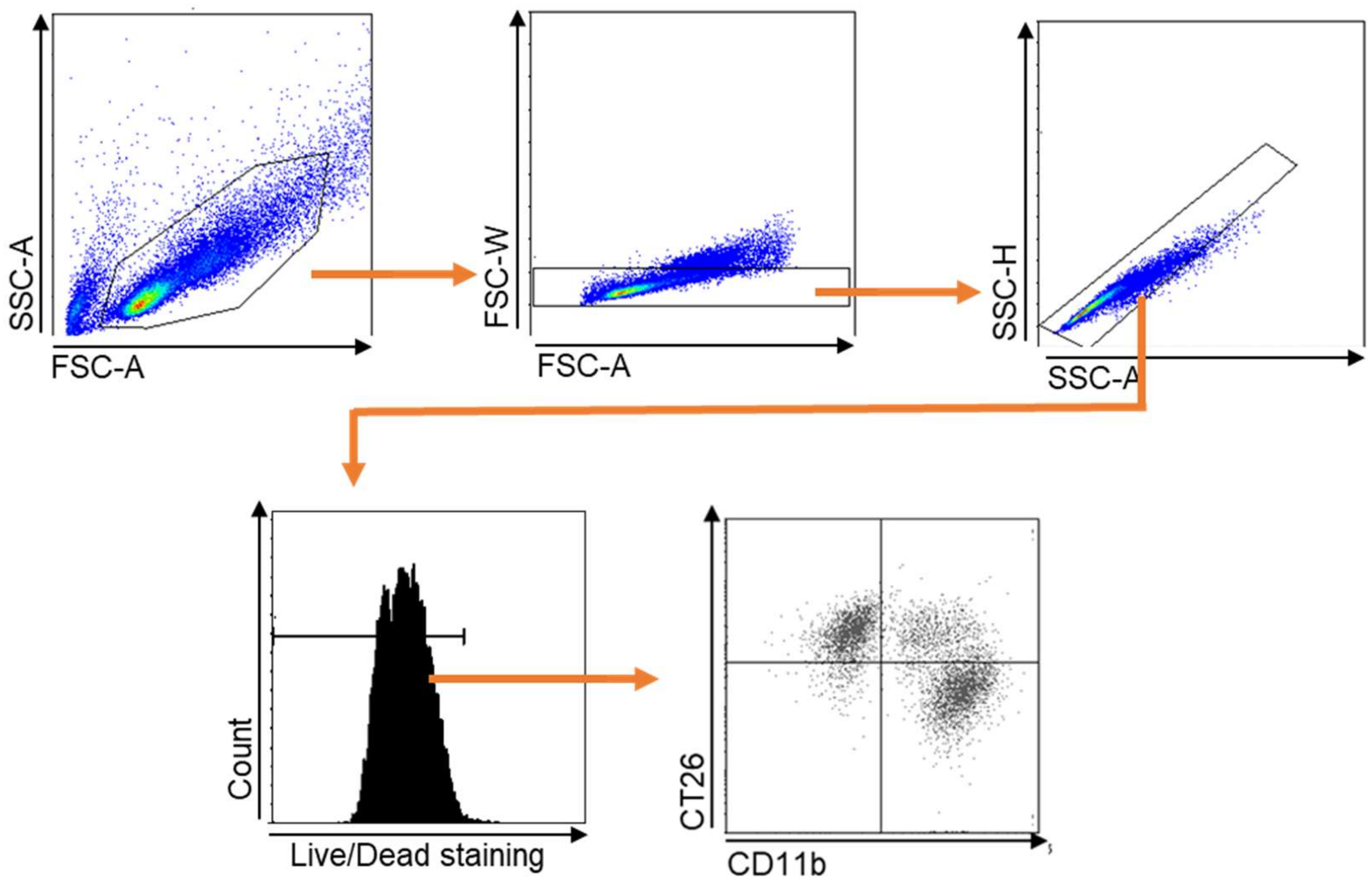

Supplemental Fig. 2: Representative images of FACS analysis demonstrate the gating strategy for phagocytosis of CT26 in RAW264.7 cells treated with CT26-CM.

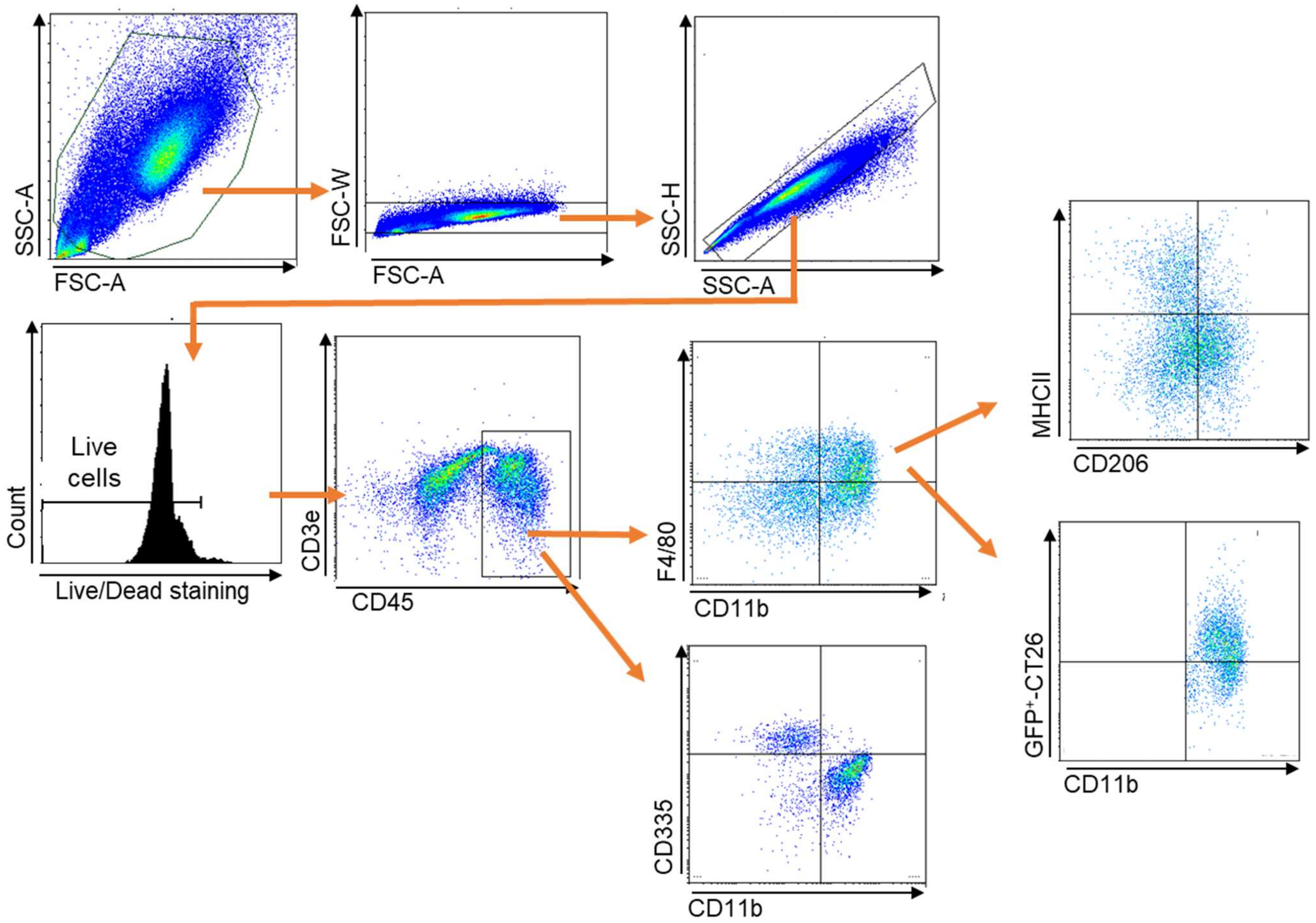

Supplemental Fig. 3: Representative images of FACS analysis demonstrate the gating strategy for macrophages and NK cells in tumor-infiltrating leukocytes isolated from implanted tumors.

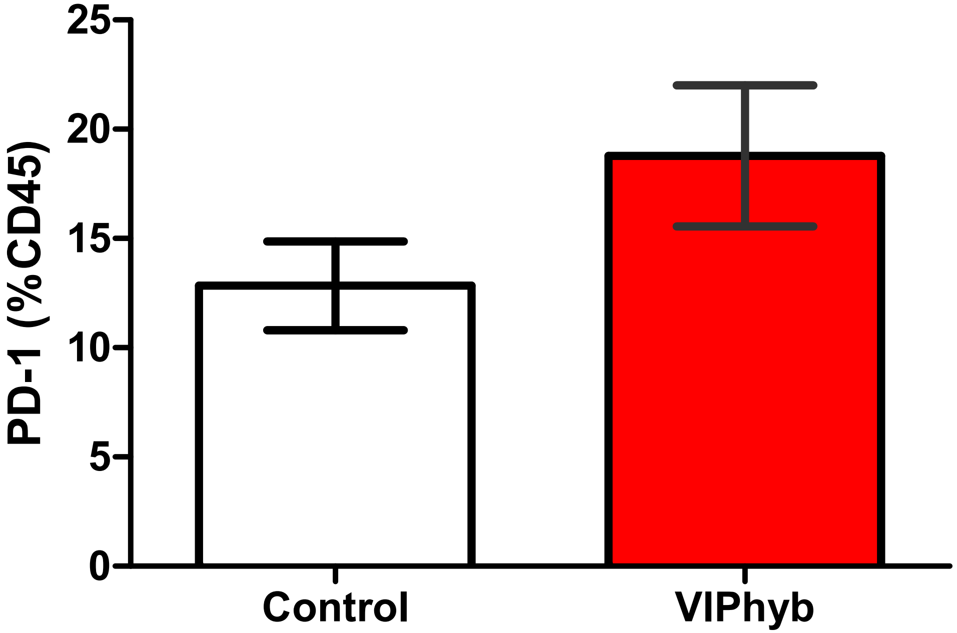

Supplemental Fig. 4: Blocking VIP signaling tended to increase PD-1-expressing macrophage populations in implanted tumors after each treatment in SCID mice. Flow cytometric analysis of PD-1$^+$ macrophages in TILs isolated from control and VIP hybrid groups (n=4–5/group).

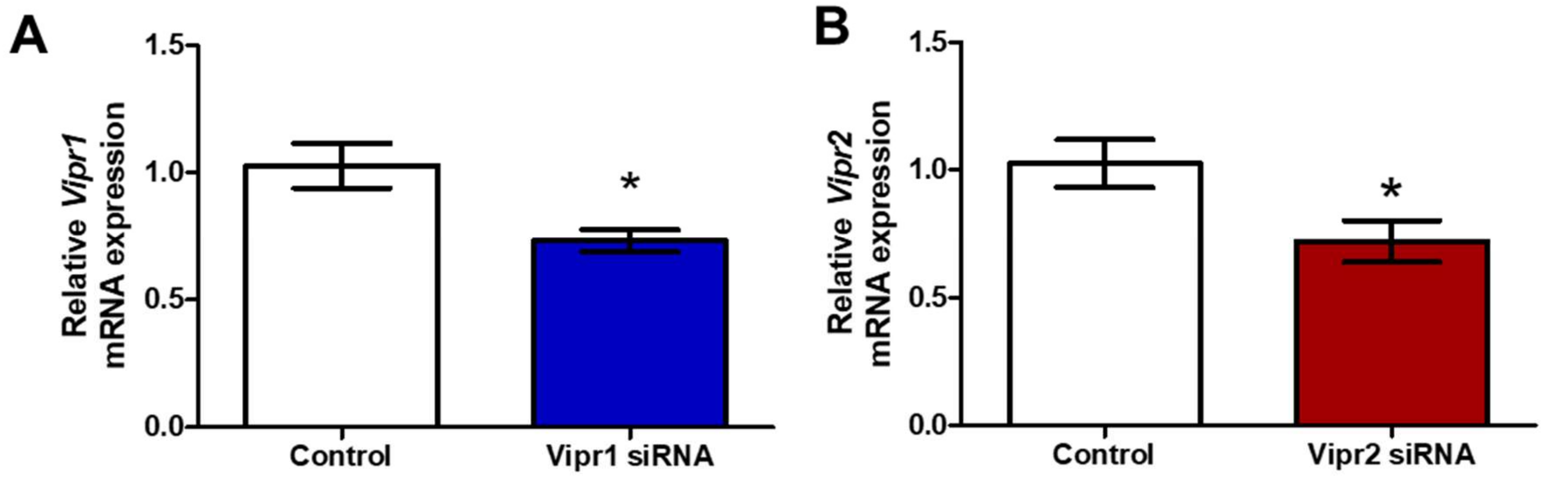

Supplemental Fig. 5: Effect of *Vipr1* or *Vipr2* siRNA on gene expression of *Vipr1* (A) or *Vipr2* (B), respectively. Negative control siRNA was used as control. *$p<0.05$ vs control.

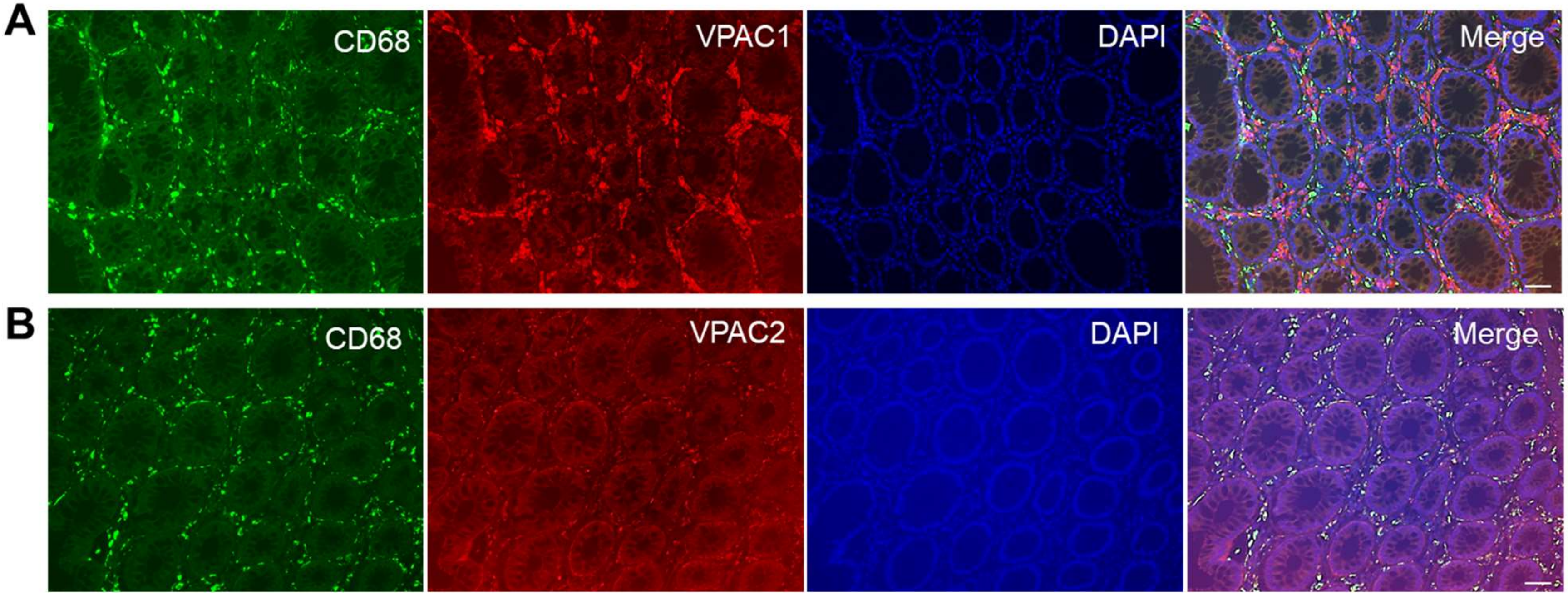

Supplemental Fig. 6: Representative images of CD68 (green) and VPAC1 (red; A); and CD68 (green) and VPAC2 (red; B) staining in normal adjacent tissue of human colorectal cancer specimens. Nuclei were stained by DAPI (blue). Scale bar=100μm.
